# Supplementary material for: Mapping of quantitative trait loci for traits linked to fusarium head blight in barley
Source: PLoS One. 2020 Feb 4;15(2):e0222375. doi: 10.1371/journal.pone.0222375 (PMC6999892; doi:10.1371/journal.pone.0222375)
Supplement: S1 Fig — (DOCX) [file pone.0222375.s001.docx]

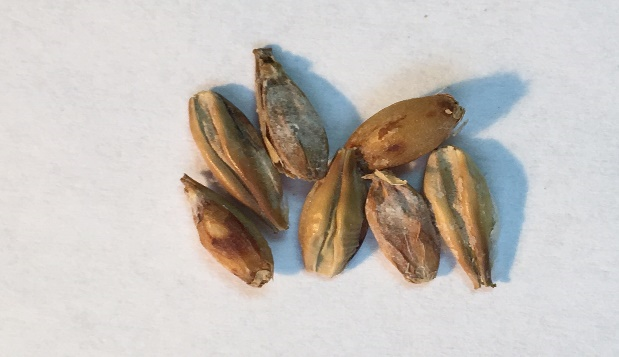


**S1 Fig. Seeds, observed in LCam plants, with moderate or severe *Fusarium* symptoms. Seeds are thin, with some dark discolouration. This image was captured at 40 x magnification under the Motic BA410-E microscope.**
