# Supplementary material for: Mapping of quantitative trait loci for traits linked to fusarium head blight in barley
Source: PLoS One. 2020 Feb 4;15(2):e0222375. doi: 10.1371/journal.pone.0222375 (PMC6999892; doi:10.1371/journal.pone.0222375)
Supplement: S2 Fig — (DOCX) [file pone.0222375.s002.docx]

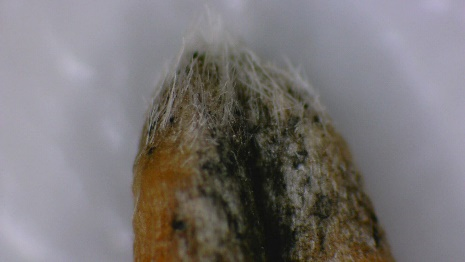


**S2 Fig. Abundant mycelial growth observed on the grain surface. This image was captured at 40 × magnification under the Motic BA410-E microscope.**
