# Supplementary material for: Mapping of quantitative trait loci for traits linked to fusarium head blight in barley
Source: PLoS One. 2020 Feb 4;15(2):e0222375. doi: 10.1371/journal.pone.0222375 (PMC6999892; doi:10.1371/journal.pone.0222375)
Supplement: S3 Fig — (DOCX) [file pone.0222375.s003.docx]

2H

1H

**S3 Fig. The positions of QTLs (chromosomes 1H and 2H) detected for studied traits.**

QFHB.IPG-2H_3

QDen.IPG-2H_3, QLSt.IPG-2H_2, QGWS.IPG-2H_2, QTGW.IPG-2H_2

QSte.IPG-2H_2

QNSS.IPG-2H_2

QDen.IPG-2H_4

QDen.IPG-2H_2

QFHB.IPG-2H_2

QTGW.IPG-2H_1

QFHB.IPG-2H_1

QLS.IPG-2H, QLSt.IPG-2H_1, QHLKn.IPG-2H_2

QNSS.IPG-2H_1, QNGS.IPG-2H, QDen.IPG-2H_1, QGWS.IPG-2H_1, QGY.IPG-2H, QHD.IPG-2H, QHLKw.IPG-2H

QNSS.IPG-1H_1, QNGS.IPG-1H_1, QLS.IPG-1H, QGY.IPG-1H, QLSt.IPG-1H

QSte.IPG-2H_1

QHLKn.IPG-2H_1

QNGS.IPG-1H_2

QNSS.IPG-1H_2

SCRI_RS_174051

BOPA1_5537-283

BOPA1_8065-1203

SCRI_RS_230497

BOPA2_12_10937

SCRI_RS_129821

BOPA1_4331-1475

BOPA1_ABC12560-1-1-421

BOPA1_5880-2547

BK_13

BK_12

SCRI_RS_1540307

SCRI_RS_2193337

Mb

BOPA1_4625-14137

SCRI_RS_189197

BOPA1_4625-14137

0

100

200

300

400

500

600

700

800
