# Supplementary material for: Mapping of quantitative trait loci for traits linked to fusarium head blight in barley
Source: PLoS One. 2020 Feb 4;15(2):e0222375. doi: 10.1371/journal.pone.0222375 (PMC6999892; doi:10.1371/journal.pone.0222375)
Supplement: S4 Fig — (DOCX) [file pone.0222375.s004.docx]

0

Mb

5H

4H

3H

QDen.IPG-5H_2

QLSt.IPG-3H

SCRI_RS_160471

SCRI_RS_168359

BOPA1_2533-773

SCRI_RS_151808

SCRI_RS_221814

QSte.IPG-5H_2

QNSS.IPG-3H_2

100

QGWS.IPG-4H_1

200

QSte.IPG-5H_1

QHLKn.IPG-5H

300

QTGW.IPG-4H_1

BOPA1_4342-528

QNSS.IPG-5H_2, QNGS.IPG-5H_2

QGWS.IPG-4H_2, QTGW.IPG-4H_2

SCRI_RS_165919

QGY.IPG-5H

400

SCRI_RS_206867

QHD.IPG-5H

BOPA1_3549-743

QLSt.IPG-4H_1

QGWS.IPG-5H

BOPA2_12_30533

BOPA1_2196-195

500

QLSt.IPG-4H_2

QGY.IPG-3H

QNSS.IPG-5H_1

SCRI_RS_3280

SCRI_RS_233444

QFHB.IPG-3H

BOPA1_4795-782

BOPA2_12_30239

SCRI_RS_147950

600

QNGS.IPG-5H_1, QLS.IPG-5H, QLSt.IPG-5H

SCRI_RS_235055

BOPA1_6871-945

QNSS.IPG-3H_1

QFDKn.IPG-5H

QDen.IPG-5H_1, QFHB.IPG-5H

SCRI_RS_184066

BOPA2_12_30929

BOPA1_7782-410

700

QFDKw.IPG-5H

SCRI_RS_165578

SCRI_RS_140356

800

**S4 Fig. The positions of QTLs (chromosomes 3H, 4H and 5H) detected for studied traits.**
