# Supplementary material for: Mapping of quantitative trait loci for traits linked to fusarium head blight in barley
Source: PLoS One. 2020 Feb 4;15(2):e0222375. doi: 10.1371/journal.pone.0222375 (PMC6999892; doi:10.1371/journal.pone.0222375)
Supplement: S5 Fig — (DOCX) [file pone.0222375.s005.docx]

0

7H

6H

Mb

BOPA1_2124-984

QTGW.IPG-7H_1

QFDKn.IPG-6H

SCRI_RS_115036

SCRI_RS_155654

QHLKw.IPG-7H

BOPA1_2585-2901

QFDKw.IPG-6H

100

200

300

400

QTGW.IPG-6H_2

SCRI_RS_202478

500

SCRI_RS_168994

QTGW.IPG-6H_1

SCRI_RS_168121

QFHB.IPG-7H

600

QGWS.IPG-7H_2

SCRI_RS_14491

QSte.IPG-7H

BOPA2_12_31294

700

QGWS.IPG-7H_1, QTGW.IPG-7H_2

SCRI_RS_159555

800

**S5 Fig. The positions of QTLs (chromosomes 6H and 7H) detected for studied traits.**
