# Supplementary material for: Mapping of quantitative trait loci for traits linked to fusarium head blight in barley
Source: PLoS One. 2020 Feb 4;15(2):e0222375. doi: 10.1371/journal.pone.0222375 (PMC6999892; doi:10.1371/journal.pone.0222375)
Supplement: S1 Table — (DOCX) [file pone.0222375.s006.docx]

**S1 Table. The mean values for studied traits for parental cultivars.**

| Trait | Treatment | Lubuski | | CamB | |
| --- | --- | --- | --- | --- | --- |
|  |  | mean | s.e. | mean | s.e. |
| NSS | Infection | 22.72 | 0.95 | 18.87 | 1.28 |
|  | Control | 24.89 | 0.8 | 20.36 | 1.1 |
| NGS | Infection | 21.2 | 1.05 | 16.37 | 0.98 |
|  | Control | 23.76 | 0.72 | 19.32 | 1.84 |
| LS | Infection | 7.88 | 0.49 | 7.03 | 0.32 |
|  | Control | 9.33 | 1.05 | 7.37 | 0.4 |
| Sterility | Infection | 1.077 | 0.019 | 1.162 | 0.076 |
|  | Control | 1.048 | 0.009 | 1.082 | 0.04 |
| Density | Infection | 2.913 | 0.079 | 2.683 | 0.153 |
|  | Control | 2.809 | 0.159 | 2.767 | 0.057 |
| GWS | Infection | 0.92 | 0.08 | 0.65 | 0.042 |
|  | Control | 1.176 | 0.066 | 0.869 | 0.061 |
| GY | Infection | 91.58 | 15.72 | 34.69 | 13.02 |
|  | Control | 152.96 | 6.97 | 41.94 | 5.28 |
| TGW | Infection | 42.82 | 2.45 | 41.09 | 3.66 |
|  | Control | 49.43 | 2.04 | 45.82 | 1.7 |
| HD | Infection | 158.89 | 1.35 | 147 | 0.67 |
|  | Control | 158.56 | 1.5 | 147.22 | 0.94 |
| LSt | Infection | 79.11 | 0.79 | 77.44 | 1.56 |
|  | Control | 79.89 | 1.58 | 78.78 | 1.94 |
| FHBi | Infection | 2.66 | 0.32 | 2.57 | 0.47 |
|  | Control | 1.56 | 0.39 | 1.72 | 0.43 |
| DON | Infection | 19683.89 | 821.43 | 31574 | 780.48 |
|  | Control | 119.17 | 13.58 | 268.85 | 17.42 |
| FDKn | Infection | 0.292 | 0.022 | 0.317 | 0.006 |
|  | Control | 0.179 | 0.023 | 0.152 | 0.014 |
| FDKw | Infection | 0.105 | 0.008 | 0.11 | 0.002 |
|  | Control | 0.079 | 0.004 | 0.077 | 0 |
| HLKn | Infection | 15.1 | 1.01 | 13.4 | 0.87 |
|  | Control | 22.27 | 1.05 | 19.03 | 1.93 |
| HLKw | Infection | 0.595 | 0.177 | 0.51 | 0.113 |
|  | Control | 1.258 | 0.092 | 0.825 | 0.019 |
