# Supplementary material for: Mapping of quantitative trait loci for traits linked to fusarium head blight in barley
Source: PLoS One. 2020 Feb 4;15(2):e0222375. doi: 10.1371/journal.pone.0222375 (PMC6999892; doi:10.1371/journal.pone.0222375)
Supplement: S2 Table — (DOCX) [file pone.0222375.s007.docx]

**S2 Table. The mean values for studied traits for RILs.**

| Trait | Location | Treatment | RILs | | | | |
| --- | --- | --- | --- | --- | --- | --- | --- |
|  |  |  | mean | s.e. | min. | max. | c.v. |
| NSS | LES | Infection | 23.52 | 0.15 | 16.00 | 30.20 | 11.19 |
|  |  | Control | 25.13 | 0.17 | 16.60 | 36.60 | 11.99 |
|  | NAD | Infection | 19.47 | 0.25 | 7.20 | 27.60 | 21.83 |
|  |  | Control | 22.99 | 0.11 | 16.60 | 27.60 | 8.48 |
|  | TUL | Infection | 21.35 | 0.12 | 15.60 | 27.60 | 9.82 |
|  |  | Control | 22.55 | 0.13 | 16.80 | 28.60 | 10.09 |
| NGS | LES | Infection | 22.50 | 0.15 | 15.40 | 29.90 | 11.75 |
|  |  | Control | 24.97 | 0.20 | 14.80 | 36.90 | 13.62 |
|  | NAD | Infection | 16.80 | 0.22 | 5.10 | 24.30 | 22.24 |
|  |  | Control | 21.90 | 0.12 | 15.80 | 26.80 | 9.45 |
|  | TUL | Infection | 20.14 | 0.14 | 12.80 | 26.80 | 11.93 |
|  |  | Control | 21.15 | 0.14 | 13.00 | 27.80 | 11.63 |
| LS | LES | Infection | 8.08 | 0.07 | 5.45 | 11.79 | 14.36 |
|  |  | Control | 8.78 | 0.07 | 5.86 | 15.70 | 14.33 |
|  | NAD | Infection | 6.77 | 0.05 | 4.40 | 12.44 | 11.79 |
|  |  | Control | 7.84 | 0.03 | 6.47 | 9.59 | 7.27 |
|  | TUL | Infection | 7.16 | 0.04 | 5.67 | 10.11 | 8.86 |
|  |  | Control | 7.45 | 0.04 | 6.06 | 10.91 | 9.11 |
| Sterility | LES | Infection | 1.046 | 0.002 | 1.004 | 1.208 | 2.643 |
|  |  | Control | 1.011 | 0.004 | 0.720 | 1.263 | 6.256 |
|  | NAD | Infection | 1.161 | 0.003 | 0.990 | 1.412 | 4.143 |
|  |  | Control | 1.051 | 0.002 | 0.965 | 1.169 | 2.553 |
|  | TUL | Infection | 1.064 | 0.003 | 1.009 | 1.531 | 5.144 |
|  |  | Control | 1.069 | 0.003 | 0.996 | 1.431 | 4.199 |
| Density | LES | Infection | 2.929 | 0.011 | 2.456 | 3.540 | 6.674 |
|  |  | Control | 2.888 | 0.020 | 1.439 | 4.265 | 11.891 |
|  | NAD | Infection | 2.880 | 0.034 | 1.012 | 3.925 | 20.133 |
|  |  | Control | 2.935 | 0.012 | 2.421 | 3.424 | 6.845 |
|  | TUL | Infection | 2.988 | 0.013 | 2.406 | 3.576 | 7.733 |
|  |  | Control | 3.031 | 0.013 | 2.472 | 3.536 | 7.372 |
| GWS | LES | Infection | 0.932 | 0.010 | 0.100 | 1.419 | 18.609 |
|  |  | Control | 1.099 | 0.013 | 0.059 | 1.701 | 19.776 |
|  | NAD | Infection | 0.711 | 0.016 | 0.184 | 1.453 | 38.876 |
|  |  | Control | 1.125 | 0.008 | 0.648 | 1.485 | 13.031 |
|  | TUL | Infection | 0.935 | 0.008 | 0.501 | 1.355 | 15.130 |
|  |  | Control | 1.060 | 0.009 | 0.678 | 1.537 | 14.836 |
| GY | LES | Infection | 40.99 | 1.61 | 1.27 | 179.70 | 67.26 |
|  |  | Control | 75.29 | 2.54 | 5.04 | 212.34 | 6.56 |
|  | NAD | Infection | - | - | - | - | - |
|  |  | Control | 128.91 | 1.82 | 38.69 | 226.64 | 24.49 |
|  | TUL | Infection | 95.57 | 1.67 | 24.06 | 174.98 | 30.20 |
|  |  | Control | 122.22 | 2.16 | 19.89 | 217.66 | 30.63 |
| TGW | LES | Infection | 41.24 | 0.27 | 4.22 | 51.12 | 11.51 |
|  |  | Control | 43.98 | 0.38 | 2.44 | 56.80 | 14.90 |
|  | NAD | Infection | 42.27 | 0.76 | 19.00 | 72.00 | 30.87 |
|  |  | Control | 51.29 | 0.21 | 36.00 | 61.95 | 7.22 |
|  | TUL | Infection | 46.41 | 0.24 | 22.88 | 70.77 | 8.97 |
|  |  | Control | 50.02 | 0.20 | 38.89 | 64.43 | 6.88 |
| HD | LES | Infection | 158.15 | 0.22 | 148.00 | 165.00 | 2.38 |
|  |  | Control | 158.46 | 0.22 | 149.00 | 165.00 | 2.38 |
|  | NAD | Infection | 153.16 | 0.18 | 145.00 | 160.00 | 2.01 |
|  |  | Control | 153.47 | 0.18 | 146.00 | 160.00 | 2.00 |
|  | TUL | Infection | 155.30 | 0.19 | 146.00 | 165.00 | 2.10 |
|  |  | Control | 155.25 | 0.17 | 148.00 | 162.00 | 1.93 |
| LSt | LES | Infection | 70.62 | 0.48 | 53.00 | 102.00 | 11.79 |
|  |  | Control | 77.67 | 0.38 | 60.00 | 95.00 | 8.53 |
|  | NAD | Infection | 82.70 | 0.28 | 70.00 | 96.00 | 5.82 |
|  |  | Control | 83.47 | 0.25 | 65.00 | 95.00 | 5.12 |
|  | TUL | Infection | 79.36 | 0.29 | 65.00 | 91.00 | 6.38 |
|  |  | Control | 79.71 | 0.33 | 62.00 | 92.00 | 7.16 |
| FHBi | LES | Infection | 1.89 | 0.04 | 0.69 | 4.11 | 33.13 |
|  |  | Control | 0.62 | 0.04 | 0.00 | 2.40 | 112.11 |
|  | NAD | Infection | 2.17 | 0.04 | 0.00 | 3.89 | 34.69 |
|  |  | Control | 0.99 | 0.05 | 0.00 | 2.89 | 88.97 |
|  | TUL | Infection | 2.26 | 0.04 | 0.69 | 3.89 | 31.50 |
|  |  | Control | 0.96 | 0.05 | 0.00 | 2.83 | 94.97 |
| DON | LES | Infection | 26439.47 | 344.24 | 8512.00 | 39990.00 | 22.51 |
|  |  | Control | 67.98 | 4.26 | 0.00 | 300.15 | 108.62 |
|  | NAD | Infection | 25684.27 | 347.71 | 8062.00 | 39980.00 | 23.45 |
|  |  | Control | 85.47 | 5.33 | 0.00 | 300.25 | 107.96 |
|  | TUL | Infection | 27144.47 | 330.11 | 9909.00 | 39990.00 | 21.06 |
|  |  | Control | 76.39 | 4.73 | 0.00 | 302.12 | 106.71 |
| FDKn | LES | Infection | - | - | - | - | - |
|  |  | Control | - | - | - | - | - |
|  | NAD | Infection | 0.289 | 0.002 | 0.110 | 0.381 | 14.515 |
|  |  | Control | 0.181 | 0.002 | 0.069 | 0.277 | 20.451 |
|  | TUL | Infection | - | - | - | - | - |
|  |  | Control | - | - | - | - | - |
| FDKw | LES | Infection | - | - | - | - | - |
|  |  | Control | - | - | - | - | - |
|  | NAD | Infection | 0.103 | 0.001 | 0.074 | 0.143 | 12.321 |
|  |  | Control | 0.083 | 0.000 | 0.070 | 0.106 | 8.080 |
|  | TUL | Infection | - | - | - | - | - |
|  |  | Control | - | - | - | - | - |
| HLKn | LES | Infection | - | - | - | - | - |
|  |  | Control | - | - | - | - | - |
|  | NAD | Infection | 15.59 | 0.16 | 0.00 | 21.90 | 17.96 |
|  |  | Control | 20.87 | 0.15 | 12.10 | 29.30 | 12.10 |
|  | TUL | Infection | - | - | - | - | - |
|  |  | Control | - | - | - | - | - |
| HLKw | LES | Infection | - | - | - | - | - |
|  |  | Control | - | - | - | - | - |
|  | NAD | Infection | 0.628 | 0.016 | 0.119 | 1.349 | 44.604 |
|  |  | Control | 1.095 | 0.009 | 0.617 | 1.453 | 13.684 |
|  | TUL | Infection | - | - | - | - | - |
|  |  | Control | - | - | - | - | - |
