# Supplementary material for: Mapping of quantitative trait loci for traits linked to fusarium head blight in barley
Source: PLoS One. 2020 Feb 4;15(2):e0222375. doi: 10.1371/journal.pone.0222375 (PMC6999892; doi:10.1371/journal.pone.0222375)
Supplement: S3 Table — (DOCX) [file pone.0222375.s008.docx]

**S3 Table. ANOVA results, variance components and heritability estimated for studied traits.**

| Trait | P-values for effects of | | | Variance components and std. errors for | | | | | | | | Heritability | |
| --- | --- | --- | --- | --- | --- | --- | --- | --- | --- | --- | --- | --- | --- |
|  |  |  |  |  |  |  |  |  |  |  |  |  |  |
|  | location | treatment | location × treatment interacton | lines | s.e. | interaction line × location | s.e. | interaction line × treatment | s.e. | interaction line × location × treatment | s.e. |  |  |
|  |  |  |  |  |  |  |  |  |  |  |  | inoculated | un-inoculated |
| NSS | <0.001 | <0.001 | <0.001 | 2.447* | 0.397 | 0.32 | 0.135 | 0.02 | 0.093 | 0 | - | 0.69 | 0.8887 |
| NGS | <0.001 | <0.001 | <0.001 | 2.766* | 0.446 | 0.394 | 0.143 | 0.033 | 0.096 | 0 | - | 0.71 | 0.8814 |
| LS | <0.001 | <0.001 | <0.001 | 0.2643* | 0.0443 | 0.0634 | 0.0178* | 0 | - | 0.0181 | 0.0194 | 0.72 | 0.7916 |
| Sterility | <0.001 | <0.001 | <0.001 | 0.000139 | 0.00005 | 0.000251 | 0.000065* | 0 | - | 0 | - | - | 0.4839 |
| Density | <0.001 | 0.138 | 0.009 | 0.0256* | 0.00448 | 0.0056 | 0.00176* | 0.0022 | 0.00135 | 0 | - | 0.74 | 0.8174 |
| GWS | <0.001 | <0.001 | <0.001 | 0.00969* | 0.00167 | 0.00144 | 0.0065 | 0.00065 | 0.00052 | 0 | - | 0.59 | 0.8764 |
| GY | <0.001 | <0.001 | 0.006 | 329.9* | 52.8 | 0 | - | 0 | - | 0 | - | 0.63 | 0.7533 |
| TGW | <0.001 | <0.001 | <0.001 | 1.9 | 0.87 | 1.22 | 1.03 | 0.97 | 0.88 | 0 | - | 0.06 | 0.72 |
| HD | <0.001 | <0.001 | 0.035 | 9.625* | 1.419 | 1.142 | 0.134* | 0.013 | 0.024 | 0.019 | 0.041 | 0.92 | 0.92 |
| LSt | <0.001 | <0.001 | <0.001 | 9.55* | 2.047 | 5.118 | 1.414* | 0.519 | 0.881 | 11.234* | 1.425 | 0.58 | 0.61 |
| FHBi | <0.001 | <0.001 | 0.02 | 0.0471 | 0.0325 | 0.4351 | 0.0461* | 0.0045 | 0.0037 | 0.0187* | 0.0056 | 0.14 | 0.29 |
| DON_x_ | <0.001 | - | - | 21271008* | 3204801 | 0 | - | - | - | - | - | - | - |
| FDKn | - | <0.001 | - | 0.000228 | 0.000082 | - | - | 0.000189 | 0.000084 | - | - | - | - |
| FDKw | - | <0.001 | - | 0.0000157 | 0.00000595 | - | - | 0.00001913* | 0.00000611 | - | - | - | - |
| HLKn | - | <0.001 | - | 2.568 | 0.494 | - | - | 0.248 | 0.256 | - | - | - | - |
| HLKw | - | <0.001 | - | 0.00645 | 0.00231 | - | - | 0.00161 | 0.00244 | - | - | - | - |

* variance component at least three times greater than its standard error

s.e.- standard error

_x-_ ANOVA analysis only for infection condition
