# Supplementary material for: Mapping of quantitative trait loci for traits linked to fusarium head blight in barley
Source: PLoS One. 2020 Feb 4;15(2):e0222375. doi: 10.1371/journal.pone.0222375 (PMC6999892; doi:10.1371/journal.pone.0222375)
Supplement: S4 Table — (DOCX) [file pone.0222375.s009.docx]

**S4 Table. QTLs identified in the LCam population for the observed traits.**

| Trait | QTL ID | Chr | Position (cM) | Nearest marker | Distance to the nearest marker | Marker sequence start in reference genome (bp) | Marker sequence end in reference genome (bp) | -log_10_ (P-value) | QTL×E (0 = NO, 1 = YES) | Significant additive effect | | | | | | Percent of variance explained by QTL in given conditions (%) | | | | | |
| --- | --- | --- | --- | --- | --- | --- | --- | --- | --- | --- | --- | --- | --- | --- | --- | --- | --- | --- | --- | --- | --- |
|  |  |  |  |  |  |  |  |  |  | AND | | NAD | | TUL | | AND | | NAD | | TUL | |
|  |  |  |  |  |  |  |  |  |  | infection | control | infection | control | infection | control | infection | control | infection | control | infection | control |
| NSS | QNSS.IPG-1H_1 | 1H | 0.00 | BOPA1_4625-1413 | 0.00 | 522486880 | 522487120 | 6.27 | 0 | -0.84 | -0.84 | -0.84 | -0.84 | -0.84 | -0.84 | 13.64 | 14.57 | 11.45 | 22.81 | 22.90 | 18.61 |
| NGS | QNGS.IPG-1H_1 | 1H | 0.00 | BOPA1_4625-1413 | 0.00 | 522486880 | 522487120 | 2.88 | 0 | -0.49 | -0.49 | -0.49 | -0.49 | -0.49 | -0.49 | 5.03 | 3.86 | 4.65 | 6.90 | 6.22 | 5.35 |
| LS | QLS.IPG-1H | 1H | 0.00 | BOPA1_4625-1413 | 0.00 | 522486880 | 522487120 | 13.57 | 1 | -0.59 | -0.55 | -0.18 | -0.36 | -0.37 | -0.40 | 35.58 | 33.15 | 12.54 | 56.20 | 49.40 | 48.11 |
| GY | QGY.IPG-1H | 1H | 0.00 | BOPA1_4625-1413 | 0.00 | 522486880 | 522487120 | 2.87 | 0 | -5.59 | -5.59 |  | -5.59 | -5.59 | -5.59 | 9.69 | 3.98 |  | 5.78 | 8.84 | 5.24 |
| LSt | QLSt.IPG-1H | 1H | 0.00 | BOPA1_4625-1413 | 0.00 | 522486880 | 522487120 | 8.57 | 0 | -1.98 | -1.98 | -1.98 | -1.98 | -1.98 | -1.98 | 5.65 | 9.50 | 20.88 | 32.36 | 21.85 | 17.12 |
| NGS | QNGS.IPG-1H_2 | 1H | 98.00 | SCRI_RS_189197 | -1.60 | 35727343 | 35727463 | 3.28 | 0 | -0.52 | -0.52 | -0.52 | -0.52 | -0.52 | -0.52 | 5.52 | 4.24 | 5.11 | 7.59 | 6.84 | 5.88 |
| NSS | QNSS.IPG-1H_2 | 1H | 232.40 | SCRI_RS_201086 | 0.00 | 523222032 | 523222033 | 4.23 | 1 |  | 0.51 |  | 0.41 |  |  | 0.10 | 5.37 | 0.14 | 5.51 | 1.04 | 1.38 |
| HLKn | QHLKn.IPG-2H_1 | 2H | 0.00 | SCRI_RS_219333 | 0.00 | 35977 | 35978 | 3.57 | 0 |  |  | 0.56 | 0.56 |  |  |  |  | 7.51 | 6.81 |  |  |
| Sterility | QSte.IPG-2H_1 | 2H | 17.80 | SCRI_RS_154030 | 0.00 | 22687872 | 22687873 | 41.25 | 1 |  |  |  | 0.02 | 0.03 | 0.03 | 5.73 | 6.91 | 2.49 | 68.47 | 77.57 | 122.82 |
| LS | QLS.IPG-2H | 2H | 21.00 | BK_13 | 0.00 | 29126530 | 29126531 | 18.79 | 1 | -0.77 | -0.57 | -0.17 | -0.29 | -0.27 | -0.31 | 60.85 | 36.02 | 10.85 | 37.16 | 26.10 | 29.52 |
| LSt | QLSt.IPG-2H_1 | 2H | 21.00 | BK_13 | 0.00 | 29126530 | 29126531 | 14.16 | 1 | -4.80 | -4.15 | -2.02 | -1.62 | -2.60 | -1.78 | 33.17 | 41.68 | 21.62 | 21.53 | 37.52 | 13.88 |
| HLKn | QHLKn.IPG-2H_2 | 2H | 21.00 | BK_13 | 0.00 | 29126530 | 29126531 | 26.34 | 0 |  |  | -1.96 | -1.96 |  |  |  |  | 92.79 | 84.06 |  |  |
| NSS | QNSS.IPG-2H_1 | 2H | 22.00 | BK_12 | 0.00 | 29125743 | 29125744 | 31.33 | 1 | -1.92 | -1.34 |  | -1.45 | -1.32 | -1.46 | 71.81 | 37.38 | 4.79 | 67.98 | 56.95 | 56.58 |
| NGS | QNGS.IPG-2H | 2H | 22.00 | BK_12 | 0.00 | 29125743 | 29125744 | 44.66 | 1 | -2.24 | -2.02 | -0.72 | -2.01 | -1.99 | -2.14 | 103.77 | 65.14 | 9.88 | 115.41 | 101.99 | 101.02 |
| Density | QDen.IPG-2H_1 | 2H | 22.00 | BK_12 | 0.00 | 29125743 | 29125744 | 15.17 | 1 |  |  |  | -0.10 | -0.10 | -0.09 | 2.10 | 0.42 | 0.72 | 31.43 | 22.31 | 17.77 |
| GWS | QGWS.IPG-2H_1 | 2H | 22.00 | BK_12 | 0.00 | 29125743 | 29125744 | 16.49 | 0 | -0.09 | -0.09 | -0.09 | -0.09 | -0.09 | -0.09 | 41.61 | 31.74 | 36.18 | 46.27 | 57.92 | 44.50 |
| GY | QGY.IPG-2H | 2H | 22.00 | BK_12 | 0.00 | 29125743 | 29125744 | 10.59 | 0 | -10.75 | -10.75 |  | -10.75 | -10.75 | -10.75 | 35.81 | 14.71 |  | 21.37 | 32.68 | 19.36 |
| HD | QHD.IPG-2H | 2H | 22.00 | BK_12 | 0.00 | 29125743 | 29125744 | 31.90 | 0 | -2.74 | -2.74 | -2.74 | -2.74 | -2.74 | -2.74 | 57.11 | 60.07 | 82.80 | 85.33 | 76.31 | 87.87 |
| HLKw | QHLKw.IPG-2H | 2H | 22.00 | BK_12 | 0.00 | 29125743 | 29125744 | 7.56 | 0 |  |  | -0.08 | -0.08 |  |  |  |  | 22.14 | 33.27 |  |  |
| FHBi | QFHB.IPG-2H_1 | 2H | 23.10 | BOPA1_5880-2547 | 0.00 | 31559962 | 31559963 | 7.56 | 1 | 0.21 | 0.23 | 0.16 |  | 0.32 | 0.48 | 12.26 | 14.59 | 5.09 | 3.69 | 22.48 | 30.68 |
| TGW | QTGW.IPG-2H_1 | 2H | 87.20 | BOPA1_ABC12560-1-1-421 | 0.00 | 96162710 | 96162711 | 4.41 | 1 | -1.20 |  |  | -0.87 |  |  | 11.41 | 0.83 | 1.98 | 8.40 | 0.02 | 2.14 |
| FHBi | QFHB.IPG-2H_2 | 2H | 87.70 | BOPA1_4331-1475 | 0.00 | 100641976 | 100641977 | 3.44 | 1 |  |  |  |  | -0.17 | -0.28 | 0.07 | 0.01 | 0.94 | 1.09 | 6.65 | 10.66 |
| Density | QDen.IPG-2H_2 | 2H | 113.90 | BOPA1_8065-1203 | 0.00 | 631976772 | 631976773 | 10.45 | 0 | 0.07 | 0.07 | 0.07 | 0.07 | 0.07 | 0.07 | 15.91 | 8.95 | 4.29 | 14.82 | 11.09 | 11.12 |
| Sterility | QSte.IPG-2H_2 | 2H | 197.40 | SCRI_RS_230497 | 5.60 | 746332765 | 746332766 | 3.59 | 1 | 0.00 |  |  |  |  | 0.01 | 10.78 | 1.27 | 2.03 | 1.43 | 0.84 | 13.41 |
| FHBi | QFHB.IPG-2H_3 | 2H | 216.70 | SCRI_RS_129821 | 0.00 | 766143691 | 766143692 | 8.14 | 0 | 0.21 | 0.21 | 0.21 | 0.21 | 0.21 | 0.21 | 11.71 | 11.56 | 8.02 | 6.52 | 9.55 | 5.81 |
| Density | QDen.IPG-2H_3 | 2H | 225.26 | BOPA2_12_10937 | -3.44 | 754651630 | 754651631 | 3.29 | 1 | 0.03 |  |  |  |  | 0.03 | 2.81 | 0.90 | 0.01 | 0.42 | 0.03 | 2.48 |
| LSt | QLSt.IPG-2H_2 | 2H | 225.26 | BOPA2_12_10937 | -3.44 | 754651630 | 754651631 | 3.43 | 0 | 0.99 | 0.99 | 0.99 | 0.99 | 0.99 | 0.99 | 1.40 | 2.36 | 5.19 | 8.04 | 5.43 | 4.25 |
| GWS | QGWS.IPG-2H_2 | 2H | 228.70 | BOPA2_12_10937 | 0.00 | 754651630 | 754651631 | 4.39 | 1 |  | 0.04 |  | 0.04 |  | 0.04 | 0.00 | 6.85 | 2.06 | 8.35 | 0.01 | 7.13 |
| TGW | QTGW.IPG-2H_2 | 2H | 228.70 | BOPA2_12_10937 | 0.00 | 754651630 | 754651631 | 4.04 | 1 |  |  |  | 0.95 |  | 0.51 | 0.38 | 0.43 | 0.74 | 9.96 | 0.37 | 4.39 |
| NSS | QNSS.IPG-2H_2 | 2H | 229.80 | SCRI_RS_174051 | 0.00 | 753586515 | 753586516 | 4.21 | 1 |  | 0.66 |  | 0.30 |  | 0.49 | 0.09 | 9.10 | 0.66 | 3.01 | 0.21 | 6.41 |
| Density | QDen.IPG-2H_4 | 2H | 290.50 | BOPA1_5537-283 | 0.00 | 699187393 | 699187394 | 8.30 | 0 | 0.06 | 0.06 | 0.06 | 0.06 | 0.06 | 0.06 | 11.58 | 6.52 | 3.13 | 10.78 | 8.07 | 8.10 |

| Trait | QTL ID | Chr | Position (cM) | Nearest marker | Distance to the nearest marker | Marker sequence start in reference genome (bp) | Marker sequence end in reference genome (bp) | -log_10_ (P-value) | QTL×E (0 = NO, 1 = YES) | Significant additive effect | | | | | | Percent of variance explained by QTL in given conditions (%) | | | | | |
| --- | --- | --- | --- | --- | --- | --- | --- | --- | --- | --- | --- | --- | --- | --- | --- | --- | --- | --- | --- | --- | --- |
|  |  |  |  |  |  |  |  |  |  | AND | | NAD | | TUL | | AND | | NAD | | TUL | |
|  |  |  |  |  |  |  |  |  |  | infection | control | infection | control | infection | control | infection | control | infection | control | infection | control |
| GY | QGY.IPG-3H | 3H | 90.20 | SCRI_RS_147950 | 0.00 | 592563494 | 592563495 | 2.38 | 0 | -3.67 | -3.67 |  | -3.67 | -3.67 | -3.67 | 4.18 | 1.72 |  | 2.49 | 3.81 | 2.26 |
| FHBi | QFHB.IPG-3H | 3H | 114.00 | BOPA1_6871-945 | -3.40 | 617523850 | 617523851 | 6.27 | 0 | 0.18 | 0.18 | 0.18 | 0.18 | 0.18 | 0.18 | 8.38 | 8.27 | 5.73 | 4.66 | 6.83 | 4.15 |
| NSS | QNSS.IPG-3H_1 | 3H | 166.20 | BOPA1_7782-410 | -5.70 | 687549675 | 687549676 | 3.25 | 0 | -0.51 | -0.51 | -0.51 | -0.51 | -0.51 | -0.51 | 5.10 | 5.45 | 4.28 | 8.52 | 8.56 | 6.95 |
| NSS | QNSS.IPG-3H_2 | 3H | 234.23 | SCRI_RS_151808 | -7.57 | 18879955 | 18879956 | 10.61 | 0 | 1.04 | 1.04 | 1.04 | 1.04 | 1.04 | 1.04 | 21.22 | 22.67 | 17.81 | 35.48 | 35.62 | 28.95 |
| LSt | QLSt.IPG-3H | 3H | 241.80 | SCRI_RS_221814 | 0.00 | 18619104 | 18619105 | 5.33 | 0 | 1.17 | 1.17 | 1.17 | 1.17 | 1.17 | 1.17 | 1.97 | 3.31 | 7.27 | 11.27 | 7.61 | 5.96 |
| GWS | QGWS.IPG-4H_1 | 4H | 15.30 | BOPA1_2533-773 | 0.00 | 5074747 | 5074748 | 6.02 | 1 | 0.02 | 0.06 |  | 0.03 | 0.03 | 0.03 | 2.48 | 13.81 | 4.08 | 5.62 | 8.79 | 6.57 |
| TGW | QTGW.IPG-4H_1 | 4H | 115.60 | BOPA1_3549-743 | 0.00 | 569760181 | 569760182 | 4.52 | 1 | -1.15 |  |  | -0.78 |  |  | 10.60 | 0.08 | 2.88 | 6.68 | 1.59 | 0.05 |
| GWS | QGWS.IPG-4H_2 | 4H | 127.40 | BOPA1_2196-195 | 0.00 | 585200008 | 585200009 | 3.86 | 1 | -0.04 |  |  | -0.03 |  |  | 8.60 | 2.97 | 2.74 | 5.42 | 0.23 | 0.46 |
| TGW | QTGW.IPG-4H_2 | 4H | 127.40 | BOPA1_2196-195 | 0.00 | 585200008 | 585200009 | 3.17 | 0 | -0.89 | -0.89 | -0.89 | -0.89 | -0.89 | -0.89 | 6.32 | 5.10 | 1.50 | 8.71 | 9.69 | 13.19 |
| LSt | QLSt.IPG-4H_1 | 4H | 143.45 | SCRI_RS_233444 | 1.15 | 604747857 | 604747858 | 3.40 | 1 | -2.05 | -1.54 | 0.82 |  |  |  | 6.03 | 5.75 | 3.54 | 0.00 | 2.70 | 1.04 |
| LSt | QLSt.IPG-4H_2 | 4H | 212.48 | BOPA2_12_30239 | -1.72 | 645030797 | 645030798 | 4.36 | 1 |  | -2.21 |  |  | 1.11 |  | 0.48 | 11.76 | 0.06 | 2.84 | 6.86 | 0.02 |
| FDKw | QFDKw.IPG-5H | 5H | 87.80 | SCRI_RS_140356 | 0.00 | 606929329 | 606929449 | 9.04 | 0 |  |  | 0.00 | 0.00 |  |  |  |  | 7.35 | 26.61 |  |  |
| FDKn | QFDKn.IPG-5H | 5H | 89.50 | SCRI_RS_165578 | 1.10 | 606312041 | 606312042 | 6.99 | 0 |  |  | -0.01 | -0.01 |  |  |  |  | 13.29 | 19.01 |  |  |
| Density | QDen.IPG-5H_1 | 5H | 93.90 | SCRI_RS_184066 | 0.00 | 601143767 | 601143768 | 5.57 | 1 | 0.06 | 0.08 |  |  | 0.03 | 0.03 | 12.83 | 12.73 | 1.70 | 0.35 | 2.06 | 1.76 |
| FHBi | QFHB.IPG-5H | 5H | 95.60 | SCRI_RS_184066 | 1.70 | 601143767 | 601143768 | 6.96 | 1 |  |  | -0.34 | -0.37 | -0.29 | -0.24 | 5.38 | 1.05 | 21.66 | 21.25 | 18.95 | 8.12 |
| NGS | QNGS.IPG-5H_1 | 5H | 97.30 | BOPA2_12_30929 | 0.00 | 599134827 | 599134828 | 14.67 | 1 | -1.24 | -0.56 |  |  | -0.41 | -0.51 | 31.86 | 4.94 | 0.15 | 1.63 | 4.35 | 5.71 |
| LS | QLS.IPG-5H | 5H | 97.30 | BOPA2_12_30929 | 0.00 | 599134827 | 599134828 | 14.46 | 1 | -0.59 | -0.31 |  | -0.11 | -0.17 | -0.22 | 35.35 | 10.87 | 4.15 | 4.96 | 10.64 | 14.36 |
| LSt | QLSt.IPG-5H | 5H | 97.30 | BOPA2_12_30929 | 0.00 | 599134827 | 599134828 | 8.37 | 1 | -4.38 | -2.35 | -1.20 |  | -1.40 | -1.04 | 27.65 | 13.36 | 7.67 | 2.72 | 10.86 | 4.71 |
| NSS | QNSS.IPG-5H_1 | 5H | 98.67 | SCRI_RS_235055 | 1.37 | 598322835 | 598322836 | 16.95 | 1 | -1.28 | -0.37 |  |  |  | -0.42 | 32.10 | 2.78 | 0.26 | 1.18 | 2.27 | 4.61 |
| GWS | QGWS.IPG-5H | 5H | 100.03 | BOPA1_4795-782 | -1.37 | 597797236 | 597797237 | 8.66 | 1 | -0.07 | -0.07 | -0.03 | -0.03 | -0.03 | -0.04 | 27.01 | 21.65 | 5.58 | 5.20 | 6.11 | 10.30 |
| Density | QDen.IPG-5H_2 | 5H | 157.50 | SCRI_RS_168359 | 0.00 | 3438902 | 3438903 | 2.39 | 0 | 0.03 | 0.03 | 0.03 | 0.03 | 0.03 | 0.03 | 4.01 | 2.26 | 1.08 | 3.73 | 2.79 | 2.80 |
| Sterility | QSte.IPG-5H_1 | 5H | 205.80 | BOPA1_4342-528 | 0.00 | 482426682 | 482426683 | 11.02 | 1 | 0.01 |  |  |  | -0.02 |  | 25.92 | 0.54 | 0.20 | 0.02 | 24.45 | 2.68 |
| Sterility | QSte.IPG-5H_2 | 5H | 229.11 | SCRI_RS_160471 | -5.19 | 31837938 | 31837939 | 8.91 | 1 |  |  |  | 0.01 | 0.02 | 0.02 | 11.63 | 11.13 | 11.62 | 12.68 | 38.56 | 34.48 |
| HD | QHD.IPG-5H | 5H | 268.60 | SCRI_RS_3280 | 0.00 | 558186475 | 558186476 | 2.43 | 0 | 0.51 | 0.51 | 0.51 | 0.51 | 0.51 | 0.51 | 1.94 | 2.04 | 2.82 | 2.90 | 2.60 | 2.99 |
| GY | QGY.IPG-5H | 5H | 285.20 | BOPA2_12_30533 | 0.00 | 541555119 | 541555120 | 3.52 | 0 | -5.22 | -5.22 |  | -5.22 | -5.22 | -5.22 | 8.43 | 3.46 |  | 5.03 | 7.70 | 4.56 |
| NSS | QNSS.IPG-5H_2 | 5H | 286.90 | SCRI_RS_206867 | 1.10 | 540972424 | 540972425 | 3.61 | 0 | -0.43 | -0.43 | -0.43 | -0.43 | -0.43 | -0.43 | 3.62 | 3.87 | 3.04 | 6.05 | 6.08 | 4.94 |
| NGS | QNGS.IPG-5H_2 | 5H | 286.90 | SCRI_RS_206867 | 1.10 | 540972424 | 540972425 | 3.45 | 1 | -0.47 | -0.68 |  | -0.34 |  |  | 4.60 | 7.40 | 2.70 | 3.20 | 0.59 | 1.15 |
| HLKn | QHLKn.IPG-5H | 5H | 288.00 | SCRI_RS_165919 | 0.00 | 533523885 | 533523886 | 2.71 | 0 |  |  | -0.45 | -0.45 |  |  |  |  | 4.99 | 4.52 |  |  |

| Trait | QTL ID | Chr | Position (cM) | Nearest marker | Distance to the nearest marker | Marker sequence start in reference genome (bp) | Marker sequence end in reference genome (bp) | -log_10_ (P-value) | QTL×E (0 = NO, 1 = YES) | Significant additive effect | | | | | | Percent of variance explained by QTL in given conditions (%) | | | | | |
| --- | --- | --- | --- | --- | --- | --- | --- | --- | --- | --- | --- | --- | --- | --- | --- | --- | --- | --- | --- | --- | --- |
|  |  |  |  |  |  |  |  |  |  | AND | | NAD | | TUL | | AND | | NAD | | TUL | |
|  |  |  |  |  |  |  |  |  |  | infection | control | infection | control | infection | control | infection | control | infection | control | infection | control |
| TGW | QTGW.IPG-6H_1 | 6H | 19.33 | SCRI_RS_168121 | 5.33 | 560541712 | 560541831 | 3.39 | 1 |  | -1.05 | -2.25 |  |  |  | 0.98 | 7.13 | 9.55 | 2.26 | 0.40 | 0.41 |
| TGW | QTGW.IPG-6H_2 | 6H | 29.30 | SCRI_RS_202478 | 0.00 | 545710168 | 545710169 | 3.10 | 0 | 0.83 | 0.83 | 0.83 | 0.83 | 0.83 | 0.83 | 5.56 | 4.49 | 1.32 | 7.67 | 8.53 | 11.61 |
| FDKw | QFDKw.IPG-6H | 6H | 84.80 | SCRI_RS_155654 | 0.00 | 40874748 | 40874749 | 1.74 | 0 |  |  | 0.00 | 0.00 |  |  |  |  | 1.13 | 4.09 |  |  |
| FDKn | QFDKn.IPG-6H | 6H | 98.33 | SCRI_RS_115036 | -1.37 | 34026843 | 34026844 | 1.81 | 1 |  |  |  | 0.01 |  |  |  |  | 0.00 | 9.70 |  |  |
| HLKw | QHLKw.IPG-7H | 7H | 2.70 | BOPA1_2585-2901 | 0.00 | 47582498 | 47582499 | 4.76 | 1 |  |  | -0.06 |  |  |  |  |  | 13.25 | 1.24 |  |  |
| TGW | QTGW.IPG-7H_1 | 7H | 15.00 | BOPA1_2124-984 | 0.00 | 33030625 | 33030626 | 7.60 | 0 | 1.06 | 1.06 | 1.06 | 1.06 | 1.06 | 1.06 | 8.93 | 7.21 | 2.12 | 12.32 | 13.71 | 18.66 |
| GWS | QGWS.IPG-7H_1 | 7H | 119.80 | SCRI_RS_159555 | 0.00 | 623889305 | 623889306 | 4.51 | 1 |  |  | -0.06 |  |  |  | 2.30 | 1.31 | 14.47 | 0.28 | 1.35 | 1.70 |
| TGW | QTGW.IPG-7H_2 | 7H | 119.80 | SCRI_RS_159555 | 0.00 | 623889305 | 623889306 | 4.09 | 1 | -1.21 | -1.08 |  |  |  | -0.59 | 11.70 | 7.49 | 2.97 | 0.30 | 3.28 | 5.70 |
| FHBi | QFHB.IPG-7H | 7H | 172.10 | SCRI_RS_168994 | 0.00 | 590233720 | 590233721 | 3.80 | 1 |  |  | -0.17 |  |  |  | 2.93 | 0.01 | 5.53 | 2.21 | 0.67 | 2.05 |
| GWS | QGWS.IPG-7H_2 | 7H | 215.40 | SCRI_RS_14491 | 0.00 | 593837517 | 593837518 | 3.45 | 1 |  | -0.05 |  |  |  |  | 0.07 | 10.29 | 1.87 | 0.31 | 0.45 | 2.45 |
| Sterility | QSte.IPG-7H | 7H | 221.33 | BOPA2_12_31294 | 5.33 | 594218014 | 594218134 | 2.39 | 0 | -0.003 | -0.003 | -0.003 | -0.003 | -0.003 | -0.003 | 6.90 | 1.06 | 1.52 | 3.44 | 0.84 | 1.26 |
