# Supplementary material for: Mapping of quantitative trait loci for traits linked to fusarium head blight in barley
Source: PLoS One. 2020 Feb 4;15(2):e0222375. doi: 10.1371/journal.pone.0222375 (PMC6999892; doi:10.1371/journal.pone.0222375)
Supplement: S5 Table — (DOCX) [file pone.0222375.s010.docx]

**S5 Table. GO overrepresentation analysis in regions B, E and F containing QTL for FHB-related traits.**

| Region | GO term-ID | p-value | x | n | X | N | Description | Genes in test set |
| --- | --- | --- | --- | --- | --- | --- | --- | --- |
| B | | | | | | | | |
| Molecular function | | | | | | | | |
|  | 30976 | 7.1495E-6 | 3 | 12 | 61 | 18670 | thiamin pyrophosphate binding | HORVU2HR1G014390\|HORVU2HR1G013170\|HORVU2HR1G014360 |
|  | 15018 | 9.2728E-6 | 3 | 13 | 61 | 18670 | galactosylgalactosylxylosylprotein 3-beta-glucuronosyltransferase activity | HORVU2HR1G013520\|HORVU2HR1G013630\|HORVU2HR1G013590 |
|  | 15020 | 1.1774E-5 | 3 | 14 | 61 | 18670 | glucuronosyltransferase activity | HORVU2HR1G013520\|HORVU2HR1G013630\|HORVU2HR1G013590 |
|  | 4650 | 3.2382E-4 | 3 | 41 | 61 | 18670 | polygalacturonase activity | HORVU1HR1G079170\|HORVU1HR1G079140\|HORVU1HR1G079130 |
| E | | | | | | | | |
| Molecular function | | | | | | | | |
|  | 5249 | 6.3049E-7 | 4 | 22 | 59 | 18670 | voltage-gated potassium channel activity | HORVU5HR1G096440\|HORVU5HR1G095550\|HORVU5HR1G095590\| HORVU5HR1G095540 |
|  | 22843 | 7.6143E-7 | 4 | 23 | 59 | 18670 | voltage-gated cation channel activity | HORVU5HR1G096440\|HORVU5HR1G095550\|HORVU5HR1G095590\| HORVU5HR1G095540 |
|  | 5267 | 1.4949E-6 | 4 | 27 | 59 | 18670 | potassium channel activity | HORVU5HR1G096440\|HORVU5HR1G095550\|HORVU5HR1G095590\| HORVU5HR1G095540 |
|  | 5261 | 2.0136E-6 | 4 | 29 | 59 | 18670 | cation channel activity | HORVU5HR1G096440\|HORVU5HR1G095550\|HORVU5HR1G095590\| HORVU5HR1G095540 |
|  | 22832 | 1.7136E-5 | 4 | 49 | 59 | 18670 | voltage-gated channel activity | HORVU5HR1G096440\|HORVU5HR1G095550\|HORVU5HR1G095590\| HORVU5HR1G095540 |
|  | 5244 | 1.7136E-5 | 4 | 49 | 59 | 18670 | voltage-gated ion channel activity | HORVU5HR1G096440\|HORVU5HR1G095550\|HORVU5HR1G095590\| HORVU5HR1G095540 |
|  | 4553 | 1.7968E-5 | 9 | 479 | 59 | 18670 | hydrolase activity, hydrolyzing O-glycosyl compounds | HORVU5HR1G095040\|HORVU5HR1G095380\|HORVU5HR1G095060\| HORVU5HR1G095220\|HORVU5HR1G095130\|HORVU5HR1G095350\| HORVU5HR1G095420\|HORVU5HR1G095100\|HORVU5HR1G095080 |
|  | 22836 | 2.5279E-5 | 4 | 54 | 59 | 18670 | gated channel activity | HORVU5HR1G096440\|HORVU5HR1G095550\|HORVU5HR1G095590\| HORVU5HR1G095540 |
|  | 16798 | 3.7454E-5 | 9 | 526 | 59 | 18670 | hydrolase activity, acting on glycosyl bonds | HORVU5HR1G095040\|HORVU5HR1G095380\|HORVU5HR1G095060\| HORVU5HR1G095220\|HORVU5HR1G095130\|HORVU5HR1G095350\| HORVU5HR1G095420\|HORVU5HR1G095100\|HORVU5HR1G095080 |
|  | 5216 | 7.0583E-5 | 4 | 70 | 59 | 18670 | ion channel activity | HORVU5HR1G096440\|HORVU5HR1G095550\|HORVU5HR1G095590\| HORVU5HR1G095540 |
|  | 22838 | 7.8825E-5 | 4 | 72 | 59 | 18670 | substrate-specific channel activity | HORVU5HR1G096440\|HORVU5HR1G095550\|HORVU5HR1G095590\| HORVU5HR1G095540 |
|  | 16758 | 1.6943E-4 | 8 | 501 | 59 | 18670 | transferase activity, transferring hexosyl groups | HORVU5HR1G096240\|HORVU5HR1G096340\|HORVU5HR1G096320\| HORVU5HR1G096390\|HORVU5HR1G095010\|HORVU5HR1G096260\| HORVU5HR1G096360\|HORVU5HR1G096310 |
|  | 22803 | 5.4485E-4 | 4 | 119 | 59 | 18670 | passive transmembrane transporter activity | HORVU5HR1G096440\|HORVU5HR1G095550\|HORVU5HR1G095590\| HORVU5HR1G095540 |
|  | 15267 | 5.4485E-4 | 4 | 119 | 59 | 18670 | channel activity | HORVU5HR1G096440\|HORVU5HR1G095550\|HORVU5HR1G095590\| HORVU5HR1G095540 |
|  | 22857 | 1.1830E-3 | 10 | 1014 | 59 | 18670 | transmembrane transporter activity | HORVU5HR1G096000\|HORVU5HR1G096440\|HORVU5HR1G095550\| HORVU5HR1G095990\|HORVU5HR1G095020\|HORVU5HR1G095030 \|HORVU5HR1G095590\|HORVU5HR1G095940\|HORVU5HR1G095820\| HORVU5HR1G095540 |
|  | 16757 | 1.7708E-3 | 8 | 716 | 59 | 18670 | transferase activity, transferring glycosyl groups | HORVU5HR1G096240\|HORVU5HR1G096340\|HORVU5HR1G096320\| HORVU5HR1G096390\|HORVU5HR1G095010\|HORVU5HR1G096260\| HORVU5HR1G096360\|HORVU5HR1G096310 |
|  | 5215 | 2.3193E-3 | 10 | 1110 | 59 | 18670 | transporter activity | HORVU5HR1G096000\|HORVU5HR1G096440\|HORVU5HR1G095550\| HORVU5HR1G095990\|HORVU5HR1G095020\|HORVU5HR1G095030 \|HORVU5HR1G095590\|HORVU5HR1G095940\|HORVU5HR1G095820\| HORVU5HR1G095540 |
|  | 8324 | 2.5902E-3 | 5 | 301 | 59 | 18670 | cation transmembrane transporter activity | HORVU5HR1G096440\|HORVU5HR1G095550\|HORVU5HR1G095030 \|HORVU5HR1G095590\|HORVU5HR1G095540 |
|  | 16695 | 3.1601E-3 | 1 | 1 | 59 | 18670 | oxidoreductase activity, acting on hydrogen as donor | HORVU5HR1G094840 |
|  | 16699 | 3.1601E-3 | 1 | 1 | 59 | 18670 | oxidoreductase activity, acting on hydrogen as donor, iron-sulfur protein as acceptor | HORVU5HR1G094840 |
|  | 43855 | 3.1601E-3 | 1 | 1 | 59 | 18670 | cyclic nucleotide-gated ion channel activity | HORVU5HR1G096440 |
|  | 4702 | 3.1601E-3 | 1 | 1 | 59 | 18670 | receptor signaling protein serine/threonine kinase activity | HORVU5HR1G095970 |
|  | 5217 | 3.1601E-3 | 1 | 1 | 59 | 18670 | intracellular ligand-gated ion channel activity | HORVU5HR1G096440 |
|  | 5221 | 3.1601E-3 | 1 | 1 | 59 | 18670 | intracellular cyclic nucleotide activated cation channel activity | HORVU5HR1G096440 |
|  | 5222 | 3.1601E-3 | 1 | 1 | 59 | 18670 | intracellular cAMP activated cation channel activity | HORVU5HR1G096440 |
|  | 8349 | 3.1601E-3 | 1 | 1 | 59 | 18670 | MAP kinase kinase kinase kinase activity | HORVU5HR1G095970 |
|  | 5057 | 3.1601E-3 | 1 | 1 | 59 | 18670 | receptor signaling protein activity | HORVU5HR1G095970 |
|  | 8901 | 3.1601E-3 | 1 | 1 | 59 | 18670 | ferredoxin hydrogenase activity | HORVU5HR1G094840 |
|  | 4338 | 3.1601E-3 | 1 | 1 | 59 | 18670 | glucan 1,3-beta-glucosidase activity | HORVU5HR1G095080 |
|  | 22834 | 6.3105E-3 | 1 | 2 | 59 | 18670 | ligand-gated channel activity | HORVU5HR1G096440 |
|  | 5242 | 6.3105E-3 | 1 | 2 | 59 | 18670 | inward rectifier potassium channel activity | HORVU5HR1G096440 |
|  | 5262 | 6.3105E-3 | 1 | 2 | 59 | 18670 | calcium channel activity | HORVU5HR1G096440 |
|  | 15276 | 6.3105E-3 | 1 | 2 | 59 | 18670 | ligand-gated ion channel activity | HORVU5HR1G096440 |
|  | 15075 | 7.9520E-3 | 5 | 393 | 59 | 18670 | ion transmembrane transporter activity | HORVU5HR1G096440\|HORVU5HR1G095550\|HORVU5HR1G095030\| HORVU5HR1G095590\|HORVU5HR1G095540 |
|  | 3979 | 9.4510E-3 | 1 | 3 | 59 | 18670 | UDP-glucose 6-dehydrogenase activity | HORVU5HR1G096370 |
|  | 4871 | 1.1764E-2 | 2 | 52 | 59 | 18670 | signal transducer activity | HORVU5HR1G095530\|HORVU5HR1G095970 |
|  | 60089 | 1.1764E-2 | 2 | 52 | 59 | 18670 | molecular transducer activity | HORVU5HR1G095530\|HORVU5HR1G095970 |
|  | 22891 | 1.1984E-2 | 5 | 435 | 59 | 18670 | substrate-specific transmembrane transporter activity | HORVU5HR1G096440\|HORVU5HR1G095550\|HORVU5HR1G095030\| HORVU5HR1G095590\|HORVU5HR1G095540 |
|  | 4674 | 1.2582E-2 | 1 | 4 | 59 | 18670 | protein serine/threonine kinase activity | HORVU5HR1G095970 |
|  | 16730 | 1.5703E-2 | 1 | 5 | 59 | 18670 | oxidoreductase activity, acting on iron-sulfur proteins as donors | HORVU5HR1G094840 |
|  | 22892 | 1.5864E-2 | 5 | 467 | 59 | 18670 | substrate-specific transporter activity | HORVU5HR1G096440\|HORVU5HR1G095550\|HORVU5HR1G095030\| HORVU5HR1G095590\|HORVU5HR1G095540 |
| Biological process | | | | | | | | |
|  | 30007 | 1.1769E-6 | 3 | 7 | 51 | 15442 | cellular potassium ion homeostasis | HORVU5HR1G095550\|HORVU5HR1G095590\|HORVU5HR1G095540 |
|  | 55075 | 4.0068E-6 | 3 | 10 | 51 | 15442 | potassium ion homeostasis | HORVU5HR1G095550\|HORVU5HR1G095590\|HORVU5HR1G095540 |
|  | 30004 | 4.0068E-6 | 3 | 10 | 51 | 15442 | cellular monovalent inorganic cation homeostasis | HORVU5HR1G095550\|HORVU5HR1G095590\|HORVU5HR1G095540 |
|  | 6875 | 2.6743E-5 | 3 | 18 | 51 | 15442 | cellular metal ion homeostasis | HORVU5HR1G095550\|HORVU5HR1G095590\|HORVU5HR1G095540 |
|  | 55067 | 3.1683E-5 | 3 | 19 | 51 | 15442 | monovalent inorganic cation homeostasis | HORVU5HR1G095550\|HORVU5HR1G095590\|HORVU5HR1G095540 |
|  | 55065 | 6.5411E-5 | 3 | 24 | 51 | 15442 | metal ion homeostasis | HORVU5HR1G095550\|HORVU5HR1G095590\|HORVU5HR1G095540 |
|  | 30003 | 8.3636E-5 | 3 | 26 | 51 | 15442 | cellular cation homeostasis | HORVU5HR1G095550\|HORVU5HR1G095590\|HORVU5HR1G095540 |
|  | 6873 | 1.2939E-4 | 3 | 30 | 51 | 15442 | cellular ion homeostasis | HORVU5HR1G095550\|HORVU5HR1G095590\|HORVU5HR1G095540 |
|  | 55082 | 3.0763E-4 | 3 | 40 | 51 | 15442 | cellular chemical homeostasis | HORVU5HR1G095550\|HORVU5HR1G095590\|HORVU5HR1G095540 |
|  | 55080 | 4.9673E-4 | 3 | 47 | 51 | 15442 | cation homeostasis | HORVU5HR1G095550\|HORVU5HR1G095590\|HORVU5HR1G095540 |
|  | 50801 | 7.8888E-4 | 3 | 55 | 51 | 15442 | ion homeostasis | HORVU5HR1G095550\|HORVU5HR1G095590\|HORVU5HR1G095540 |
|  | 16049 | 9.2140E-4 | 3 | 58 | 51 | 15442 | cell growth | HORVU5HR1G096010\|HORVU5HR1G095440\|HORVU5HR1G095910 |
|  | 8361 | 1.3413E-3 | 3 | 66 | 51 | 15442 | regulation of cell size | HORVU5HR1G096010\|HORVU5HR1G095440\|HORVU5HR1G095910 |
|  | 43062 | 1.4090E-3 | 2 | 17 | 51 | 15442 | extracellular structure organization | HORVU5HR1G096010\|HORVU5HR1G095910 |
|  | 70726 | 1.4090E-3 | 2 | 17 | 51 | 15442 | cell wall assembly | HORVU5HR1G096010\|HORVU5HR1G095910 |
|  | 10215 | 1.4090E-3 | 2 | 17 | 51 | 15442 | cellulose microfibril organization | HORVU5HR1G096010\|HORVU5HR1G095910 |
|  | 71668 | 1.4090E-3 | 2 | 17 | 51 | 15442 | plant-type cell wall assembly | HORVU5HR1G096010\|HORVU5HR1G095910 |
|  | 30198 | 1.4090E-3 | 2 | 17 | 51 | 15442 | extracellular matrix organization | HORVU5HR1G096010\|HORVU5HR1G095910 |
|  | 40007 | 1.5251E-3 | 3 | 69 | 51 | 15442 | growth | HORVU5HR1G096010\|HORVU5HR1G095440\|HORVU5HR1G095910 |
|  | 65007 | 1.9493E-3 | 19 | 2962 | 51 | 15442 | biological regulation | HORVU5HR1G096010\|HORVU5HR1G095550\|HORVU5HR1G096760 \|HORVU5HR1G096650\|HORVU5HR1G095440\|HORVU5HR1G095230\| HORVU5HR1G096590\|HORVU5HR1G095590\|HORVU5HR1G095400 \|HORVU5HR1G095410\|HORVU5HR1G095710\|HORVU5HR1G095910\| HORVU5HR1G096420\|HORVU5HR1G095540\|HORVU5HR1G095530\| HORVU5HR1G095970\|HORVU5HR1G096740\|HORVU5HR1G095630\| HORVU5HR1G096730 |
|  | 48878 | 2.0902E-3 | 3 | 77 | 51 | 15442 | chemical homeostasis | HORVU5HR1G095550\|HORVU5HR1G095590\|HORVU5HR1G095540 |
|  | 32535 | 2.2492E-3 | 3 | 79 | 51 | 15442 | regulation of cellular component size | HORVU5HR1G096010\|HORVU5HR1G095440\|HORVU5HR1G095910 |
|  | 90066 | 2.3314E-3 | 3 | 80 | 51 | 15442 | regulation of anatomical structure size | HORVU5HR1G096010\|HORVU5HR1G095440\|HORVU5HR1G095910 |
|  | 5975 | 2.5299E-3 | 10 | 1084 | 51 | 15442 | carbohydrate metabolic process | HORVU5HR1G095040\|HORVU5HR1G095380\|HORVU5HR1G095060\| HORVU5HR1G095220\|HORVU5HR1G096390\|HORVU5HR1G095130\| HORVU5HR1G095350\|HORVU5HR1G095420\|HORVU5HR1G095100\| HORVU5HR1G095080 |
|  | 65008 | 3.1130E-3 | 6 | 439 | 51 | 15442 | regulation of biological quality | HORVU5HR1G096010\|HORVU5HR1G095550\|HORVU5HR1G095440\| HORVU5HR1G095590\|HORVU5HR1G095910\|HORVU5HR1G095540 |
|  | 7263 | 3.3027E-3 | 1 | 1 | 51 | 15442 | nitric oxide mediated signal transduction | HORVU5HR1G096440 |
|  | 10540 | 3.3027E-3 | 1 | 1 | 51 | 15442 | basipetal auxin transport | HORVU5HR1G095440 |
|  | 70509 | 3.3027E-3 | 1 | 1 | 51 | 15442 | calcium ion import | HORVU5HR1G096440 |
|  | 9832 | 4.3850E-3 | 2 | 30 | 51 | 15442 | plant-type cell wall biogenesis | HORVU5HR1G096010\|HORVU5HR1G095910 |
| F | | | | | | | | |
| Molecular function | | | | | | | | |
|  | 50662 | 3.1402E-6 | 9 | 437 | 33 | 18670 | coenzyme binding | HORVU5HR1G073770\|HORVU5HR1G073900\|HORVU5HR1G073800\| HORVU5HR1G073820\|HORVU5HR1G073740\|HORVU5HR1G073960\| HORVU5HR1G073760\|HORVU5HR1G073870\|HORVU5HR1G073970 |
|  | 48037 | 8.4067E-7 | 9 | 617 | 33 | 18670 | cofactor binding | HORVU5HR1G073770\|HORVU5HR1G073900\|HORVU5HR1G073800\| HORVU5HR1G073820\|HORVU5HR1G073740\|HORVU5HR1G073960\| HORVU5HR1G073760\|HORVU5HR1G073870\|HORVU5HR1G073970 |
|  | 3854 | 7.5724E-6 | 3 | 22 | 33 | 18670 | 3-beta-hydroxy-delta5-steroid dehydrogenase activity | HORVU5HR1G073890\|HORVU5HR1G073820\|HORVU5HR1G073960 |
|  | 33764 | 7.5724E-6 | 3 | 22 | 33 | 18670 | steroid dehydrogenase activity, acting on the CH-OH group of donors, NAD or NADP as acceptor | HORVU5HR1G073890\|HORVU5HR1G073820\|HORVU5HR1G073960 |
|  | 16229 | 9.9284E-6 | 3 | 24 | 33 | 18670 | steroid dehydrogenase activity | HORVU5HR1G073890\|HORVU5HR1G073820\|HORVU5HR1G073960 |
|  | 16616 | 6.5341E-5 | 4 | 124 | 33 | 18670 | oxidoreductase activity, acting on the CH-OH group of donors, NAD or NADP as acceptor | HORVU5HR1G073890\|HORVU5HR1G073820\|HORVU5HR1G073960\| HORVU5HR1G073970 |
|  | 16614 | 1.3663E-4 | 4 | 150 | 33 | 18670 | oxidoreductase activity, acting on CH-OH group of donors | HORVU5HR1G073890\|HORVU5HR1G073820\|HORVU5HR1G073960\| HORVU5HR1G073970 |
|  | 4420 | 5.2935E-3 | 1 | 3 | 33 | 18670 | hydroxymethylglutaryl-CoA reductase (NADPH) activity | HORVU5HR1G073970 |
| Biological process | | | | | | | | |
|  | 6694 | 3.3826E-5 | 3 | 41 | 24 | 15442 | steroid biosynthetic process | HORVU5HR1G073890\|HORVU5HR1G073820\|HORVU5HR1G073960 |
|  | 8202 | 5.7988E-5 | 3 | 49 | 24 | 15442 | steroid metabolic process | HORVU5HR1G073890\|HORVU5HR1G073820\|HORVU5HR1G073960 |
