# Supplementary material for: Mapping of quantitative trait loci for traits linked to fusarium head blight in barley
Source: PLoS One. 2020 Feb 4;15(2):e0222375. doi: 10.1371/journal.pone.0222375 (PMC6999892; doi:10.1371/journal.pone.0222375)
Supplement: Attachment — Submitted filename: Plos_One.docx [file pone.0222375.s011.docx]

**Reviewer Report**

**Manuscript # PONE-D-19-23971**

Full Title: Mapping of Quantitative Trait Loci for Traits linked to Fusarium Head Blight Symptoms Evaluation in Barley RILs

**General outline of the manuscript**

The manuscript titled “Mapping of Quantitative Trait Loci for Traits linked to Fusarium Head Blight Symptoms Evaluation in Barley RILs” presented results of QTL analysis conducted for fusarium head blight (FHB) in barley. The experiments were conducted at three locations (with three replicates each) for two conditions – control and inoculated conditions (taken as treatments). The genetic mapping for QTL analysis carried out using 9K Illumina infinium arrays has led to the identification of 70 QTLs for various traits related to FHB on different chromosomes of barley.

**Comments**

Overall, the research has relevance to the identification of genetic loci controlling FHB in barley. The QTLs identified constitute an important resource for the improvement of FHB in barley. However, it still has enough room for improvement before making it available to public.

***Technical comments***: (i) The words ‘symptoms evaluation’ and ‘RILs’ can be deleted from the title (ii) It is not clear from the text whether the variance components reported in Table 2 were calculated within each location or across all the locations combined. Ideally, it would make more sense if variance components for line x location, line x treatment interactions are calculated within each location. (iii) In Table 3, the correlation coefficients were reported between FHB and traits recorded for the current study. Again, the data for individual location might be more informative than combining it across all location and subsequently using for correlation analysis. Moreover, the trait Stature was most likely recorded as an ordinal data and therefore cannot be used for calculation of correlation coefficients.

The authors must look into the fact that traits like NSS, NGS, Density, GWS and GY were negatively correlated with FHB; yet the correlation coefficient between FHB and TGW was found to be non-significant. Conducting location specific correlation analysis might point to something more important that was probably missed in the combined analysis.

Through the manuscript, the authors have been non-consistent in the usage of the names of the genotypes. The two parents have been referred to by various names like Syrian breeding line has been written as Syrian genotype, Syrian parent and CamB; and similarly Polish Cultivar has been referred to as Lubuski, European parent, European genotype and European parent genotype.

The methodology in material and methods needs to be explained a bit more. Is 20mts a standard isolation distance for fungal spores (Line 117)? The figure of concentration of inoculum on Line 124 is not clear. The number mentioned on Line 209 are not clear, as what do they refer to. The ‘LCam population’ on Line 229 is not defined in materials and methods.

Throughout the manuscript, the words ‘main QTLs’ have been used but not explained in the materials; probably, it referred to major QTLs. The words ‘trait’ and ‘character’ have been used interchangeably; the authors should have restricted to the use of one type of word only.

The discussion part is well written (requires little changes only) and results have discussed in the right context.

***Comments related to written English*:** It seems the authors have paid very little attention to the use of good written communication skills. There are numerous instances throughout the manuscript of misspelling very commonly used words like disease, classified, pollen containing, described etc (please refer to lines 21, 72, 87, 100, 305, 330, 332, 369, 383 and 388). Overall, the sentences have been written without focusing on the subject and objects of the sentences. The second paragraph on Page 4 needs to re-written. The sentence beginning on line 179 needs to be re-written. On Line 304, the sentence should begin with numbers in words only.
